# Material Design of Ultra-Thin InN/GaN Superlattices for a Long-Wavelength Light Emission

**DOI:** 10.3390/mi15030361

**Published:** 2024-03-01

**Authors:** Leilei Xiang, Enming Zhang, Wenyu Kang, Wei Lin, Junyong Kang

**Affiliations:** Department of Electronics, Department of Physics, Department of Chemistry, Tan Kah Kee Innovation Laboratory, Engineering Research Center of Micro-Nano Optoelectronic Materials, Devices at Ministry of Education, Fujian Key Laboratory of Semiconductor Materials and Application, Xiamen University, Xiamen 361005, China; 36120230156287@stu.xmu.edu.cn (L.X.); 19820221153882@stu.xmu.edu.cn (E.Z.); jykang@xmu.edu.cn (J.K.)

**Keywords:** gallium nitride heterostructure, first-principles simulation, strain regulation, charge injection

## Abstract

GaN heterostructure is a promising material for next-generation optoelectronic devices, and Indium gallium nitride (InGaN) has been widely used in ultraviolet and blue light emission. However, its applied potential for longer wavelengths still requires exploration. In this work, the ultra-thin InN/GaN superlattices (SL) were designed for long-wavelength light emission and investigated by first-principles simulations. The crystallographic and electronic properties of SL were comprehensively studied, especially the strain state of InN well layers in SL. Different strain states of InN layers were applied to modulate the bandgap of the SL, and the designed InN/GaN heterostructure could theoretically achieve photon emission of at least 650 nm. Additionally, we found the SL had different quantum confinement effects on electrons and holes, but an efficient capture of electron-hole pairs could be realized. Meanwhile, external forces were also considered. The orbital compositions of the valence band maximum (VBM) were changed with the increase in tensile stress. The transverse electric (TE) mode was found to play a leading role in light emission in normal working conditions, and it was advantageous for light extraction. The capacity of ultra-thin InN/GaN SL on long-wavelength light emission was theoretically investigated.

## 1. Introduction

InGaN is a wide bandgap semiconductor material with a tunable bandgap from 3.4 eV (GaN) to 0.7 eV (InN) [1,2]. The applications of InGaN such as light-emitting diodes (LEDs) and laser diodes (LDs) have huge potential for the virtual reality (VR) and augmented reality (AR) fields [3,4,5,6]. In these applications, InGaN is commonly used for multi-quantum wells (MQWs) to regulate the wavelength of light emission or improve the optoelectronic performance of devices. Zhou et al. utilized an InGaN quantum well with a gradually varying In content instead of that with constant In content in green LEDs. They successfully increased the light output power (LOP) of the LED from 33.9 mW to 55.2 mW at 60 A/cm^2^ and reduced the efficiency droops at 150 A/cm^2^ from 61% to 37.6% [7]. Meanwhile, Ben et al. also attempted to incorporate InGaN as quantum barrier (QB) layers in GaN-based LDs, and the slope efficiency of LDs with InGaN QB layers is 34% higher than that of LDs with GaN QB layers [8]. They attributed this great improvement in emission efficiency to the better homogeneity of the active region, which could be credited to the decreased composition pulling effect and suppression of stress between InGaN QB layers and quantum well (QW) layers. These regulations by changing the In composition and the structure of MQWs have limits. For instance, the phase separation of InN would occur in the In_x_Ga_1−x_N alloy. The published literature found that the phase separation could be suppressed by the elastic strain when the thickness of grown InGaN was less than the critical thickness (corresponding to the threshold of misfit dislocation formation) [9]. Moreover, the alloy random effect is hard to control at the atomic-level growth. Especially, InGaN alloy is easy to face phase separation. Hence, the alloy random effect needs to be avoided in the case of growth at the atomic level for quantum structures within LEDs or LDs. Encouragingly, using the InN/GaN SL to replace the InGaN MQW as an active region of optoelectronic devices does not have these two mentioned limits. The published work from our group was designed for short-wavelength light emission and grew an InN/GaN SL by metal-organic vapor phase epitaxy successfully. This SL consisted of 0.5 nm InN (i.e., 2 molecular layers) and 12 nm GaN, and it achieved an effective 390 nm emission [10,11]. Yoshikawa et al. also successfully inserted one monolayer (ML) and fractional monolayer of InN into GaN using radio frequency plasma-assisted molecular beam epitaxy (MBE), and the MQWs with one ML of InN achieved light emission at 398 nm [12]. Meanwhile, our co-workers demonstrated that the misfitted stress was able to effectively modify the bandgap of InN [13]. These research works realized light emission at short-wavelength range. However, the research for long wavelength is relatively more difficult due to the intrinsic property of the InGaN material. For InN/GaN heterostructure material design of a long-wavelength light emission, the thickness of the GaN layer needs to be reduced to decrease the misfitted stress and thus the band gap of InN. Theoretically, the quantum confinement effect of InN/GaN could be different in the case of a thinner GaN layer, which is not fully understood yet. Simultaneously, the interaction between InN and GaN layers needs to be further studied from the fundamental perspective of atomic orbital coupling.

In this work, the strain in the well layer caused by the lattice mismatch between InN and GaN layers was studied by analyzing the crystallographic configuration after full relaxation. Moreover, the origin of the strain in the well layer was fundamentally explored from the charge transfer during bonding between layers. The modulation of this internal strain to the quantum energy level of InN well layers and the changes in the quantum confinement effects on different carriers were investigated by regulating the structure of InN and GaN layers. The regulating effect of external forces induced during crystal growth on the polarization of light emitted by the InN/GaN SL was studied by artificially introducing a series of additional strains to the SL. Furthermore, the influences of charge injection while using the InN/GaN SL for the photoelectric devices on the geometry structure and band structure of SL were fully understood by introducing additional valence electrons or holes to the SL. The feasibility of using ultra-thin InN/GaN SL for a long-wavelength light emission was comprehensively discussed.

## 2. Model and Simulation Method

### 2.1. Model Establishment and Parameter Setup

The InN/GaN SL was established in the (1a×1b×nc) supercell, and the different thicknesses of InN and GaN layers were realized by changing n and substituting Ga atoms with In atoms. The Vienna Ab initio Simulation Package (VASP) based on the density functional theory (DFT) was used for first-principles simulation [14,15]. The generalized gradient approximation (GGA) in the form of Perdew–Burke–Ernzerhof (PBE) was applied for the exchange and correlation function [16]. The energy cutoff for the plane-wave basis was set as the default energy cutoff contained in the pseudopotential file, and the Brillouin zone was sampled with an 8 × 8 × 8 Monkhorst–Pack grid of k-points [17,18,19,20]. The Ga 3 d and the In 4 d electrons were also considered as valence electrons to make the results more realistic.

### 2.2. Structure Optimization and Band Simulation

The structure optimization was performed by relaxing all degrees of freedom using the conjugate gradient algorithm, where the total energy converges were within 0.1 meV. The optimized geometric and electronic structures obtained after the relaxation were applied as input for the electronic band structure calculation. To overcome the bandgap underestimation caused by using the standard DFT with GGA-PBE, the hybrid Heyd–Scuseria–Ernzerhof (HSE06) method was used to determine the more accurate bandgap [21]. Since the bandgap of InN/GaN SL was determined by the InN well layer, the value of the mixing parameter α was set as 0.25 default to evaluate the precise bandgap of the InN well layer [22]. The calculated bandgap of InN was 0.76 eV, which was close to the experimental value [13].

### 2.3. Data Processing

The differential charge density distribution was defined as:

(1) $$∆\rho =\rho_{tot}-\rho_{Ga}-\rho_{In}-\rho_{N},$$

where $\rho_{tot}$, $\rho_{Ga}$, $\rho_{In}$, and $\rho_{N}$ represent the total charge density, charge density of isolated Ga, In, and N atoms, respectively. The total charge density was obtained after the electronic self-consistency (SC) loop, and the charge density of isolated Ga, In, and N atoms was obtained by setting the maximum number of electronic SC steps to 0. The contribution of electron orbitals to the energy band was analyzed by projecting the energy band onto the electron orbitals. The band profile variation along the well plane normal in the quantum structure was revealed by arranging the projected densities of states (DOS) of each Ga-N and In-N monolayer [23]. The external forces were applied in the form of biaxial strain. A series of biaxial strains were introduced by artificially resetting the pre-relaxed lattice constants a and b and retaining atomic and lattice constant degrees of freedom along the c-axis for further relaxation. The transition dipole moment (TDM) was calculated using VASPKIT [24], and it was defined as:

(2) $$\left(P_{a\to b}\right)=\langle \psi_{a}\left|r\right|\psi_{b}\rangle =\frac{i\hbar }{\left(E_{b}-E_{a}\right)m}\langle \psi_{a}\left|P\right|\psi_{b}\rangle =\frac{i\hbar }{\left(E_{b}-E_{a}\right)}\sum_{i}C_{ai}C_{bi}G_{i},$$

where $\psi_{a}$ and $\psi_{b}$ are energy eigenstates with energy $E_{a}$ and $E_{b}$, $C_{a}$, $C_{b}$, and G are plane-wave coefficients and reciprocal space vector with the same k vector, respectively, summed over the number of plane-waves. The transition probabilities between the valence and the conduction band are revealed by the calculated sum of the squares of TDM (in a unit of Debey2). The internal charge injection in InN/GaN SL at working conditions was achieved by increasing or reducing the total valence electrons number in the structure optimization and band simulation process.

## 3. Results and Discussion

### 3.1. Crystallographic Analysis

**Figure 1:**
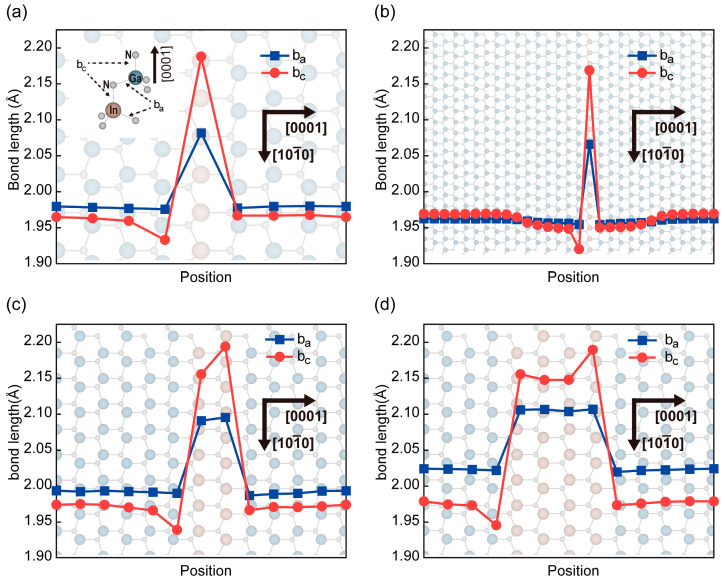
Bond length distribution along the growth direction in SL. (**a**–**d**) The distributions of bond lengths b_a_ and b_c_ along the direction [0001] in InN_1_/GaN_7_, InN_1_/GaN_27_, InN_1_/GaN_11_, and InN_4_/GaN_8_, respectively. The sub-figure in (**a**) illustrated the classification of two kinds of atomic bonds. The atomic crystal structure was the side view of the periodic extended InN_1_/GaN_7_, InN_1_/GaN_27_, InN_1_/GaN_11_, and InN_4_/GaN_8_ SL, respectively.

The atomic bonds in the InN/GaN SL were simulated, and the outcomes were compared with bonds within the bulk InN and GaN. Ⅲ-N with wurtzite structure have two types of first neighbor atomic bond length: Ⅲ-N in the basal plane and Ⅲ-N along the c-axis, and these atomic bonds were denoted as b_a_ and b_c_. The b_c_ and b_a_ of bulk compounds were calculated in advance and served as references. The values of these bonds were 1.974 Å and 1.969 Å for GaN, and 2.143 Å and 2.144 Å for InN, respectively (the difference compared with literature is less than 1%) [25]. The wurtzite GaN and InN have an anisotropic crystal structure and a nonzero spontaneous polarization along the c-axis due to the inversion symmetry center being broken and deviation from an ideal tetrahedral coordination [26]. Besides, the symmetry would be reduced due to the strain. The bond length distributions of InN_1_/GaN_7_ and InN_1_/GaN_27_ were first calculated to investigate the strains in the SL with single molecular layer InN. The length of b_a_ in the InN_1_/GaN_7_ has decreased by 3% in the InN layer, and it has increased by 0.4% in the GaN layers on average (Figure 1a). Meanwhile, the length of b_c_ has increased by 2% in the InN layer, and it has decreased by 0.5% in the GaN layers on average. This means that the InN single molecular layer was subjected to a higher in-plane compressive strain level than GaN layers, and this in-plane strain was released through the out-plane bonds by the expansion and contraction. Meanwhile, the strain in the molecular layer exacerbated the deviation of InN/GaN SLs from the ideal tetrahedral coordination and introduced piezoelectric polarization along the opposite direction in InN and GaN layers. In addition, the strain exerted by GaN layers on the InN single molecular layer should have a limit, and this limit was manifested in the SL with thick GaN layers. Therefore, the distribution of bond length in InN_1_/GaN_27_ is further illustrated in Figure 1b. The bond lengths of the GaN layer were different from that of the bulk GaN within a range of six to seven MLs on both two sides of the InN layer. The GaN layers beyond this range maintained the same bond lengths as the bulk GaN, and these layers were unable to provide strain to the InN single molecular layer. Although InN/GaN SLs with single-layer InN had been grown in different ways [11,12], the process of controlling single-layer growth of InN remains a challenge [27]. Therefore, this work also studied the internal strain of SL with multi-layer InN. The period of the SL was fixed to 12 MLs, and the thickness of InN well layers in the SL was gradually increased from one layer to four MLs. The bond lengths of InN_1_/GaN_11_ and InN_4_/GaN_8_ were taken for comparison, and that of InN_2_/GaN_10_ and InN_3_/GaN_9_ are shown in Figure S1. When the thickness of the InN well increased from one layer to four MLs, the lengths of b_a_ in each layer of InN or GaN became uniform, and the in-plane strain of GaN layers increased. Meanwhile, the changes of b_c_ were primarily concentrated at the interfaces, and other molecular layers maintained the same b_c_ as the bulk compounds. These indicated that the strains in the well and barrier layers were predominantly in-plane strains for the SL with multi-layer InN. These in-plane strains tend to accumulate within the SL and are difficult to release, thus increasing the probability of introducing dislocations during crystal growth. The changes of b_c_ at the interfaces of all structures could be explained by the charge transfer during the bonding of interfaces.

### 3.2. Electronic Properties Analysis

**Figure 2:**
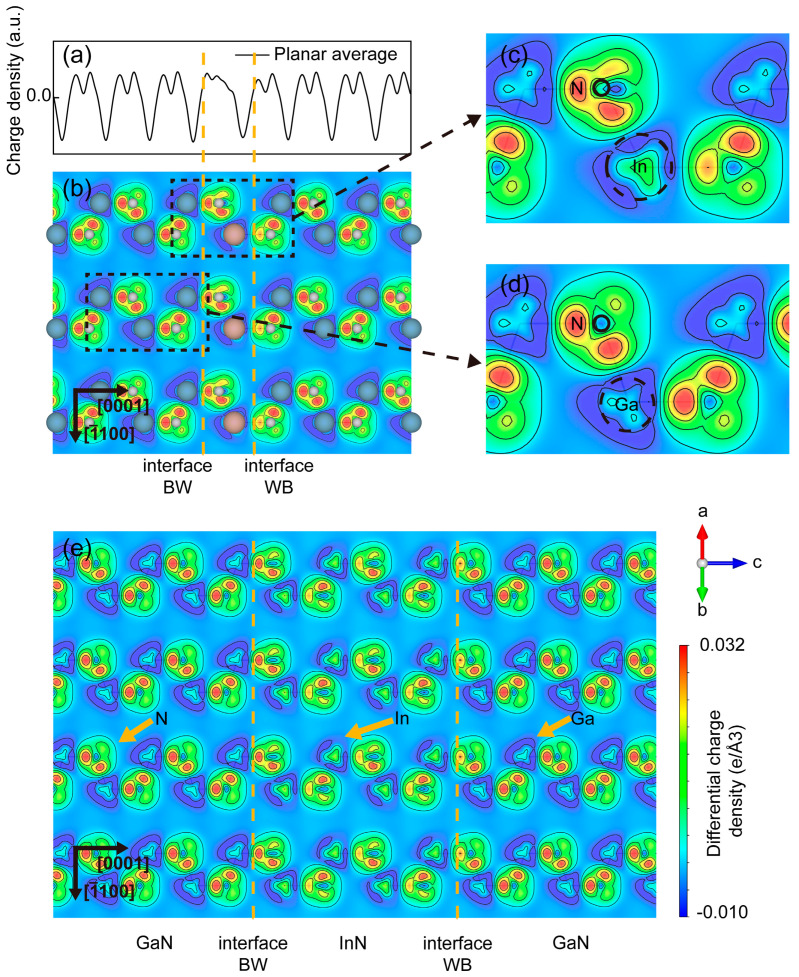
Differential charge density of InN_1_/GaN_7_ and InN_4_/GaN_8_. (**a**) The normalized planar average differential charge density of InN_1_/GaN_7_ along the c-axis. (**b**) The contour plot of the differential charge densities in a ($11\bar{2}0$) slice of InN_1_/GaN_7_. The two interfaces between the InN and the GaN layers were represented as interface BW and interface WB. (**c**,**d**) The enlarged differential charge density contour plot near the In atom and Ga atom, respectively, and the black dashed circle represented the ionic radius. (**e**) The contour plot of the differential charge densities in a ($11\bar{2}0$) slice of InN_4_/GaN_8_, and the InN and GaN areas were divided by the yellow dashed lines. The compass and the axis labels were used to calibrate the orientation of the lattice. The different colors were used to represent the positive and negative values of differential charge densities.

The differential charge density was used to evaluate the transferred charge during the bonding process in the InN/GaN SLs. The areas of electronic charge depletion and accumulation were represented by blue and red regions, respectively. The normalized planar average differential charge density is also represented in Figure 2a to quantify the bonding differences between the well and barrier layers. The contour plot map of differential charge densities in the ($11\bar{2}0$) slice of InN_1_/GaN_7_ is shown in Figure 2b, in which the b_a_ and b_c_ were included. Additionally, the areas around the In and Ga atoms were enlarged to analyze the charge transfer inside the atoms. The differential charge densities of bulk InN and GaN were also simulated as a reference to analyze the different charge transfers during the forming of the InN/GaN SL, and the results are shown in Figure S2.

The electronic charge depleted around the metal atoms accumulated between the metal and N atoms as shared charges and formed covalent bonds between the two types of atoms. In addition, this charge is primarily lost between the adjacent bonds of the metal atoms. However, a larger area of charge depletion was observed within the N atoms of the InN layer in SL by comparing Figure 2c with Figure S2a, which means the N atoms of the InN layer depleted more electrons while forming the SL. Meanwhile, more electronic charges were depleted around the In atoms compared to the bulk InN. Furthermore, the amount of charge accumulated on the b_c_ of In atoms in the SL was significantly less than that in the bulk InN, and the amount of charge accumulated on the b_a_ was comparatively more. From these charge depletions and accumulations in the InN layer, the charge transfer during the bonding process of the InN layer in InN_1_/GaN_7_ could be inferred. The electronic charges contributed by In and N atoms in the InN layer were increased. These charges tend to accumulate in the b_a_ of In atoms rather than b_c_. Hence, weakened bond strength and an increased bond length for b_c_ occurred. The increased amount of charge accumulated in the b_a_ of In atoms led to an increased bond strength and a shortened bond length. Moreover, the partial charge contributed by N atoms in the InN layer also participated in the b_c_ of the GaN layer at the interface WB, thus decreasing the bond strength and increasing the bond length.

The InN/GaN SLs with multiple-layer InN were also taken into consideration. The differential charge density of InN_4_/GaN_8_ was taken as a representative and shown in Figure 2e, and the rest of the results can be found in Figure S2c–e. It was obvious that all the N atoms in the InN layer lost more electronic charge than in the bulk compound. The amount of charge depletion in N atoms increased between the interface BW and interface WB. Meanwhile, all the In atoms depleted more charge than the bulk InN. Most of these charges accumulated in the b_a_ of each layer, thus enhancing the bond strength and reducing the bond length. Moreover, the charge accumulations in the b_c_ of the InN layers had differences, which led to the bond length of b_c_ increased on the interfaces. These changes in bond length caused by the different charge depletion and accumulation introduced the compressive strain to InN layers, which could be used to regulate the bandgap of InN layers.

### 3.3. Band Structure Analysis after the Bandgap Regulation

**Figure 3:**
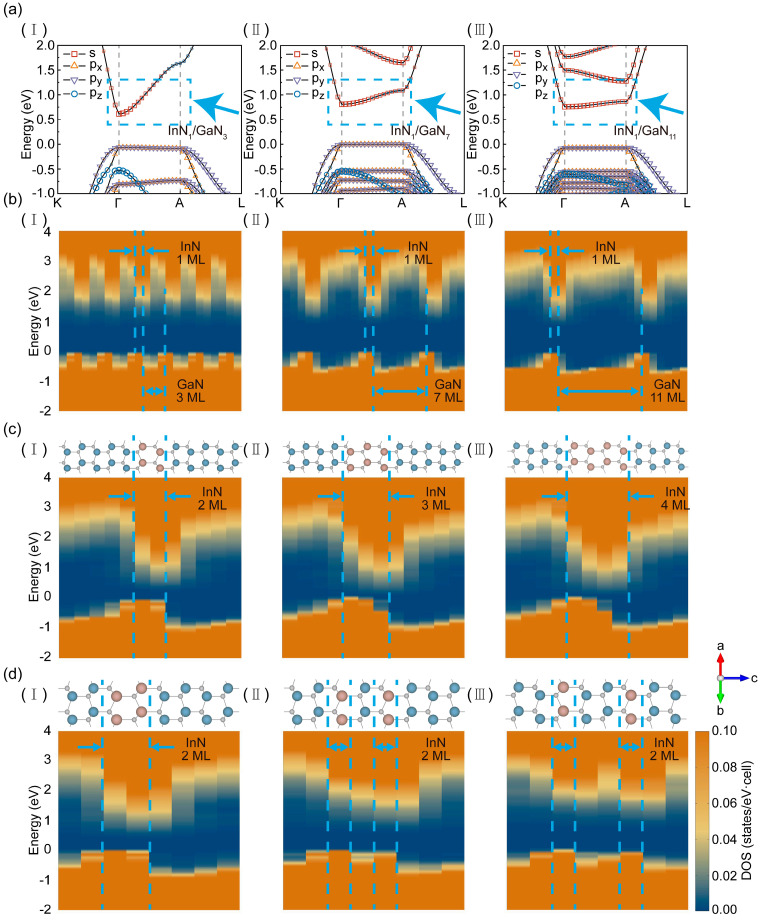
Projected density of states and band structure with different InN and GaN thickness and structures. (**a**) Band structures between K- and L-points of InN_1_/GaN_3_, InN_1_/GaN_7_, and InN_1_/GaN_11_, respectively. The blue dashed box highlighted the transition of the conduction band. (**b**) The projected densities of states of InN_1_/GaN_3_, InN_1_/GaN_7_, and InN_1_/GaN_11_. These densities of states were extended 6, 3, and 2 times along the c-axis, respectively. The blue dotted lines demarcate the InN and GaN layers in 1 period. (**c**) The projected density of states and crystal structure of the SL with different thicknesses of InN layer. The period was fixed as 12 MLs. (**d**) The band structures of the SL with different intervals of InN bilayer in the real space. The period of SL was fixed as 8 MLs. (**I**), (**II**), and (**III**) were InN bilayers with GaN intervals of 0, 1, and 2 MLs, respectively.

The feasibility of long-wavelength photons emitting by using ultra-thin InN/GaN SL was investigated by regulating the strain state of the InN layers. The strain state of the InN layers was studied in detail by changing the thickness of GaN barrier layers and the structure of InN well layers. Firstly, the regulation effect of the thickness of the GaN layer on the quantum energy level of the InN layer was analyzed (Figure 3a), and the thickness of the InN layer was maintained at one ML. The projected densities of states were used to illustrate the position, depth, and bending of the InN layer quantum well along the c-axis. With the thickness decrease in GaN (from 11 to 3 MLs), the VBM and conduction band minimum (CBM) remain fixed at the Γ-point. Simultaneously, the bandgap decreased from 0.83 eV and reached 0.78 eV when the thickness was reduced to seven MLs. A correction of bandgap was further involved by using the hybrid Heyd–Scuseria–Ernzerhof (HSE06) method [21]; 1.91 eV (i.e., around 650 nm) was gained for InN_1_/GaN_7_ and 1.82 eV (i.e., 680 nm) for InN_1_/GaN_3_. This reduction in the bandgap of the InN layer was attributed to the weakening of the strain state in the InN layer. The weakened strain state diminished the widening of the bandgap of the InN layer and made the bandgap of the InN layer closer to that of bulk InN.

The published literature about AlGaN proposes that the decrease in barrier thickness resulted in a wider bandgap due to the influence of the built-in electric field [28]. This study investigated the properties of the InN/GaN SL with single-layer InN only from the perspective of the most fundamental atomic interactions and the built-in electric field requires more investigation in the future.

Furthermore, the VBM in the InN/GaN SL was exclusively attributed to the *p_x_/p_y_* orbitals. The predominant optical polarization mode of light emitted by these orbitals was the TE mode, which was good for light extraction from SL. The valence band (VB) between the Γ-point and the A-point kept the quantum level with the thickness of GaN reduced. This means that holes were strongly localized in InN/GaN SLs even for the InN_1_/GaN_3_ SL. More significantly, the conduction band (CB) between the Γ-point and the A-point manifested as a dispersion with the thickness decrease in the GaN layer. This indicates that the electron could freely transport in the thin SL along the c-axis direction. This behavior of the electron could be explained by the configuration of barriers. The effective barrier height and width decreased after the reduction in GaN layer thickness. Consequently, this led to the coupling and hybridization of electron orbitals between the adjacent InN well layers and thus increased the electron tunneling probability and the electron injection efficiency into the SL. The different degrees of localizations of holes and electrons in SL were consistent with the results calculated by Schulz et al. in larger InGaN QWs [29]. Therefore, changing the thickness of the GaN layer could be a viable approach to achieve the emission of long-wavelength photons.

The modulation effect of the thickness of the InN layer on the band structure was also taken into consideration. The band structures of InN/GaN SL with multi-layer InN were computed by fixing the single-period thickness of the SL at 12 MLs. The band between Γ- and A-points are shown in Figure S3. The corrected band gaps of InN_2_/GaN_6_ and InN_3_/GaN_5_ were 1.31 and 1.03 eV, corresponding to the light emission of 952 and 1200 nm. The published literature indicated that the *p*-orbital component of the VBM undergoes inversion in high Al-composition AlGaN [30]. The crystal field split-off hole (CH) band contributed by the *p_z_* orbital shifted to above the heavy hole (HH) and light hole (LH) bands and formed a new VBM. However, in this study, the VBM of the InN/GaN SL with multi-layer InN was still formed by the coupling of HH and LH bands contributed by *p_y_* and *p_x_* orbitals. This indicates that the radiative recombination in the InN/GaN SL primarily emits light with TE polarization. Simultaneously, the SL with multi-layer InN had narrower bandgaps. The bandgaps obtained by using the PBE functional decreased to 0 eV when the thickness of the InN layer reached three MLs or more. It was essential to analyze the bandgap decrease from the effect of the built-in electric field within the thicker well layers. The presence of the built-in electric field can be observed in Figure 3c. It caused the band structure of the strained InN layer, which originally possessed a bandgap, to bend in real space. The band edge energies near the two interfaces eventually reached the same level, and thus resulted in the disappearance of the bandgap calculated by the PBE functional. The band bending in real space also induced the quantum-confined Stark effect within the well layers, which caused the spatial separation of electron and hole wavefunctions and carrier leakage, thus decreasing the efficiency of radiative recombination [31]. Additionally, the growth of InN/GaN SL with multi-layer InN was more prone to residual stress within the well layers, which led to the decrease in crystal quality and the introduction of non-radiative recombination centers, thus reducing the efficiency of light emission from the SL. Therefore, increasing the thickness of the InN layer to modulate the ultra-thin InN/GaN SL for a long-wavelength light emission was not an effective approach.

With the thickness increase in InN (from one to two MLs), a sharp decrease in the bandgap occurred, and it was not good for the continuous regulation of the light wavelength. This issue could be addressed potentially by increasing the separation between the two InN layers. The InN bilayer was separated by a GaN layer of varying thickness while a single-period thickness of the SL was maintained at eight MLs. The bandgap widened as the distance between the InN bilayer increased from zero to two MLs. However, a more pronounced spatial separation of electron and hole wavefunctions in real space came alongside the widened bandgap, which could further reduce the efficiency of radiative recombination. Moreover, inserting GaN layers between the InN bilayer further complicated crystal growth for the ultra-thin InN/GaN SL and made it more difficult to control the quality of crystal growth. Therefore, the method of achieving continuous bandgap tuning by inserting GaN layers between the InN bilayer was impractical. Considering the three modulation strategies mentioned above, the first method mentioned (regulation of GaN thickness) was better for achieving long-wavelength light emission. This also provides theoretical support for the high integration of InGaN full-color micro-LEDs. The factors of working conditions also need to be considered for utilizing InN/GaN SL, such as different external forces and charge injection.

### 3.4. External Forces Analysis

**Figure 4:**
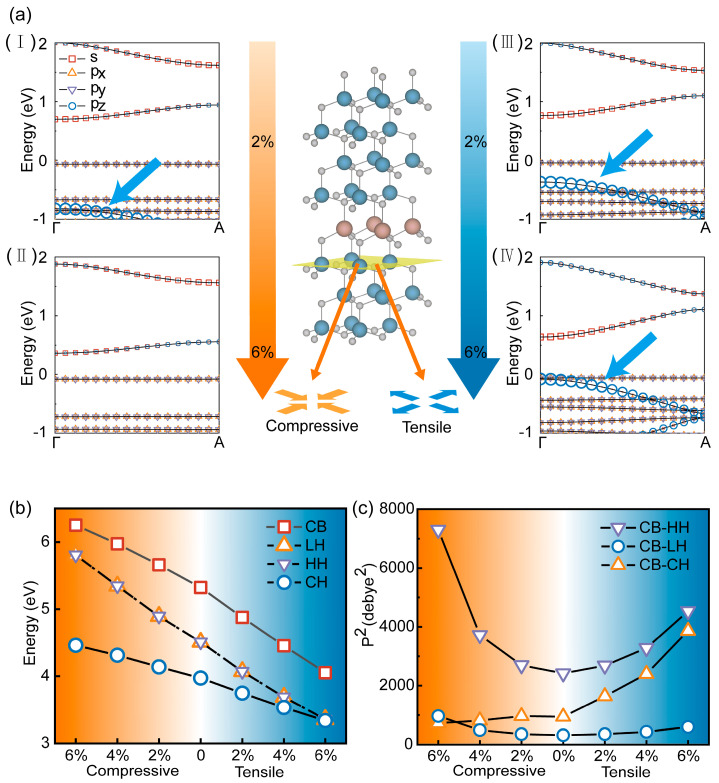
The electron structure under different external forces. (**a**) The band structures between Γ- and A-points under different external forces (2% and 6% compressive strains were represented in (**I**) and (**II**), 2% and 6% tensile strains were represented in (**III**) and (**IV**)). The blue arrow highlighted the position of the CH band and the model illustrated the form of applied strain. The contribution of different orbitals was illustrated by the size of different symbols as shown in (**I**). (**b**,**c**) Energies of different bands at Γ-point and the transition possibility between these bands.

The strain imposed by the GaN layers on the InN layer could alter the bandgap of the InN layer as previously mentioned. In the practical crystal growth, the external forces would inevitably exist in the entire SL. Therefore, a detailed investigation of the modulation effect of the external forces on the band structure of the InN/GaN SL was needed to provide theoretical guidance for utilizing the external forces. In this study, the external force in the form of the biaxial strain was applied to InN_1_/GaN_7_, and it ranged continuously from 6% tensile strain to 6% compressive strain. The quantum energy levels of the InN layer under the external forces and the transition probabilities between energy levels were investigated. The bandgap decreased slowly under the external forces as the band structures show in Figure 4a, and the position of the crystal field split-off hole (CH) band was sensitive to these forces. The CH band at the Γ-point progressively approached the VBM as the strain transformed from compressive to tensile, and it eventually crossed with the VBM when the tensile stress reached 6%. This change in the position of the energy level led to an alteration in the electron transition probability. The electron transition probability between the conduction band (CB) and the CH band increased with the rise in tensile strain, and it finally reached 3864 debye^2^. Meanwhile, the transition probability between the CB and the HH band under the same strain was 4536 debye^2^. The probabilities of electrons transitioning from CB to HH and CH bands were nearly the same. Additionally, the transverse magnetic (TM) polarized light propagating laterally (with the photoelectric field vector E parallel to the c-axis) predominated as electrons transitioned to the CH band and it was not good for light extraction [32,33]. Encouragingly, the lattice mismatch remained below 2% between the InN/GaN SL and the commonly used GaN substrate (even for the short-period InN_1_/GaN_3_) and the strain was compressive. Therefore, the InN/GaN SL had the potential for efficient light extraction.

The modulation of the external force on the position of the CH band could be explained from the perspective of the orbital coupling. The published literature extensively explored the coupling between different p orbitals in AlGaN and discussed the differences in quantum confinement effects arising from orbital coupling [34]. In this study, the CH band of the SL with single-layer InN was formed by the coupling between *p_z_* orbitals of N atoms in different molecular layers. The positive additional potential provided by *ppσ* coupling between *p_z_* orbitals was inversely proportional to the atomic distance along the c-axis. The presence of in-plane tensile strain reduced the distance between nitrogen atoms along the c-axis, which increased the additional potential and reduced the energy difference between the CH band and the Fermi energy. The stronger coupling between *p_z_* orbitals further reversed the CH band to above the HH/LH bands and formed a new VBM as the tensile strain increased to 8%. However, the position of the CH band in the SL with multi-layer InN laid deep within the valence band, and even the changes in the additional potential of orbital coupling induced by the 6% tensile strain could not reverse the gap between the CH band and the VBM. Therefore, in the SL with multi-layer InN, the TE mode consistently holds a dominant position in light emission.

### 3.5. Charge Injection Analysis

**Figure 5:**
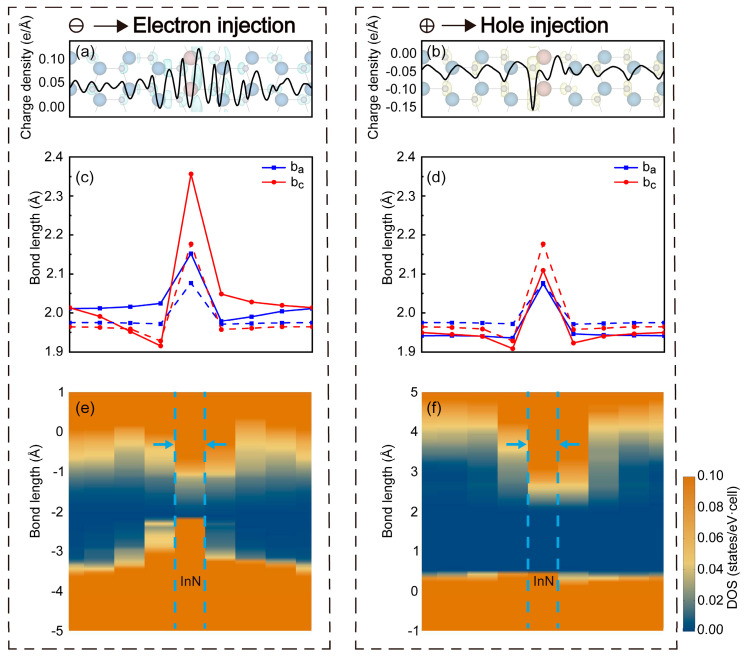
The influence of charge injection. (**a**,**b**) Distributions of injected electrons and holes along the c-axis; the blue and yellow surfaces illustrated the accumulations of these charges. (**c**,**d**) The bond length distributions. The solid lines corresponded to the SL with charge injection, and the dashed lines corresponded to the steady-state SL. (**e**,**f**) The projected density of state with charge injection. The InN layer was marked with blue dashed lines.

The photon emission in the InN/GaN SL was accompanied by the accumulation of injected charges. The distribution of injected charges within the SL and their impact on the electronic band structure was studied by introducing additional electrons or holes separately into the SL system. The results of InN_1_/GaN_7_ are shown in Figure 5.

Injected electrons and holes tended to accumulate around N atoms. Additionally, injected holes showed a higher degree of localization, but injected electrons also tended to accumulate at the WB interface and formed an electron gas (the accumulation of electron gas of InN_4_/GaN_8_ is shown in Figure S5). These were attributed to the different quantum confinement effects of the SL on the two types of carriers. These confined charge carriers participated in bonding. In comparison to the steady-state InN_1_/GaN_7_, injected electrons increased the number of bonding charges, and led to an increase in bond length and the lattice expansion. Conversely, injected holes caused a reduction in bond length and the lattice contraction. Additionally, different orbital states were occupied by the injected charges. The *s*/*p_z_* orbitals of N atoms were occupied by injected electrons, which displayed a spherical symmetry or symmetry along the c-axis. The *p_x_*/*p_y_* orbitals of N atoms were occupied by injected holes, which showed an in-plane symmetry within the c-plane. The occupation of these charges in different orbitals also altered the quantum confinement effects of the SL on charge carriers. The occupation of electrons in *s*/*p_z_* orbitals filled the conduction band and resulted in a reduction in electron potential well depth and an increase in hole potential well depth compared to the steady-state InN_1_/GaN_7_. The occupation of holes in *p_x_*/*p_y_* orbitals causes the opposite effect. From these findings, the carrier trapping behavior of the InN/GaN SL during working could be inferred. The SL inclined to capture electrons because the quantum confinement effect of SL transformed after the holes were trapped. Subsequently, the trapped electron-hole pairs would radiatively recombine and make the SL return to the original state. Therefore, the InN/GaN SL could dynamically capture the electron-hole pairs during working and it had the potential for efficient light emission.

## 4. Conclusions

This study explored the potential of ultra-thin InN/GaN SL for long-wavelength light emission by utilizing the first-principles simulations. The analysis of bond lengths within the SL and charge transfer during the bonding process revealed the origin and formation of the strain in the InN layer. The transfer of more electronic charge from In atoms and N atoms to bond b_c_ resulted in compressive in-plane strain in InN layers. The strains were further designed and modulated by changing the GaN layer thickness and combinations with different InN structures. The results demonstrated that the ultra-thin SL could emit long-wavelength light. For example, InN_1_/GaN_3_ could approach emission for 680 nm, and InN_2_/GaN_6_ for 952 nm. Additionally, this work found an efficient capture of electron-hole pairs could be realized in the InN/GaN SL, even though the SL had different quantum confinement effects on electrons and holes. At the same time, the influence of external forces was also investigated. The CH band approached the VBM as the tensile stress increased, and it eventually crossed with the VBM when the tensile stress reached 6%. Further increased tensile stress would cause the CH band to replace the HH/LH bands as the VBM. The TE mode was dominant in light emission of the SL in normal working conditions and it would be good for light extraction. This work theoretically demonstrated the potential of ultra-thin InN/GaN SL on long-wavelength light emission.

## Data Availability

Data are contained within the article.

## Supplementary Materials

The following supporting information can be downloaded at: https://www.mdpi.com/article/10.3390/mi15030361/s1. Figure S1. (a,b) The distributions of bond lengths b_a_ and b_c_ along the direction [0001] in InN_2_/GaN_10_ and InN_3_/GaN_9_, respectively. The atomic crystal structures were the side view of the periodic extended InN_2_/GaN_10_ and InN_3_/GaN_9_, respectively. Figure S2. Differential charge densities of InN, GaN, and InN/GaN SL. (a,b) were the contour plots of the differential charge densities in a ($11\bar{2}0$) slice of InN and GaN respectively. (c–e) were the contour plots of the differential charge densities in a ($11\bar{2}0$) slice of InN_1_/GaN_11_, InN_2_/GaN_10_ and InN_3_/GaN_9_ respectively. The InN and GaN areas were divided by the yellow dashed lines. The compass and the axis labels were used to calibrate the orientation of the lattice. The different colors were used to represent the positive and negative values of differential charge densities. Figure S3. (a–c) shown band structures between K and L points of InN_2_/GaN_10_, InN_3_/GaN_9_ and InN_4_/GaN_8_, respectively. Figure S4. The electronic band structure of InN_1_/GaN_7_ under 8% tensile strain. Figure S5. Distributions of injected electrons and holes. Blue and yellow surfaces represented contour planes of electrons and holes with charge density of 0.01 e/Å^3^. The InN and GaN areas were divided by the blue dashed lines.
