# Supplementary material for: Material Design of Ultra-Thin InN/GaN Superlattices for a Long-Wavelength Light Emission
Source: Micromachines (Basel). 2024 Mar 1;15(3):361. doi: 10.3390/mi15030361 (PMC10972001; doi:10.3390/mi15030361)
Supplement: Supplementary file 1 [file micromachines-15-00361-s001.zip › micromachines-2848859-supplementary.pdf]

Supplementary Material

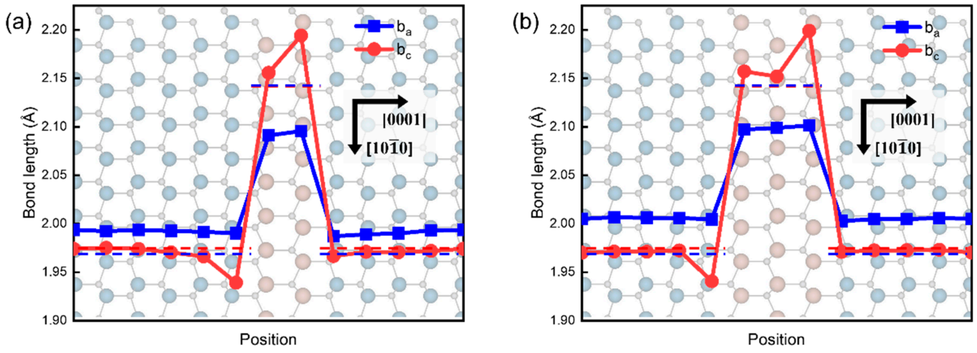

Figure S1 (**a**), and (**b**) were the distributions of bond lengths b_a and b_c along the direction [0001] in InN_2/GaN_{10}, and InN_3/GaN_9 respectively. The atomic crystal structures were the side view of the periodic extended InN_2/GaN_{10}, and InN_3/GaN_9 respectively.

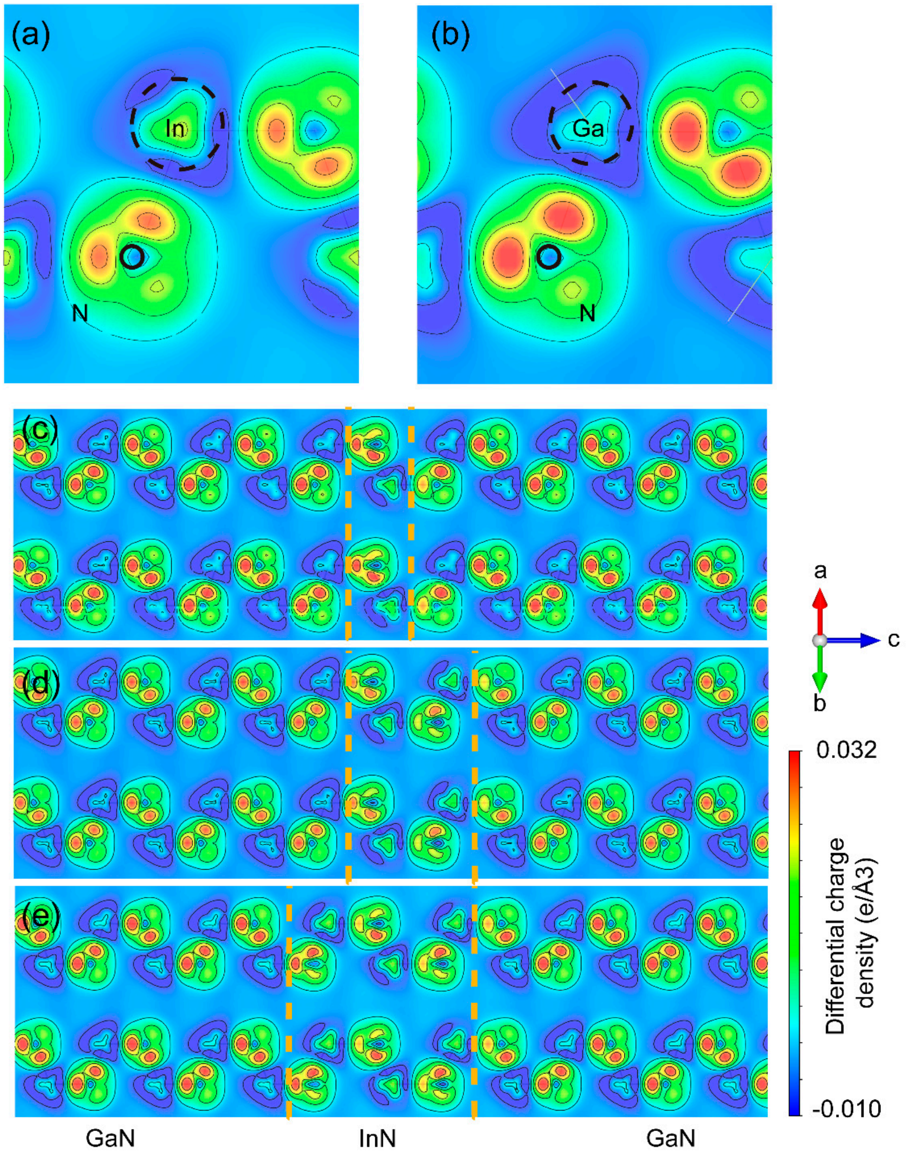

Figure S2 Differential charge densities of InN, GaN, and InN/GaN SL. (**a**) and (**b**) were the contour plots of the differential charge densities in a (11$\bar{2}$0) slice of InN and GaN respectively. (**c**), (**d**) and

(**e**) were the contour plots of the differential charge densities in a $(11\bar{2}0)$ slice of InN_1/GaN_{11}, InN_2/GaN_{10} and InN_3/GaN_9 respectively. The InN and GaN areas were divided by the yellow dashed lines. The compass and the axis labels were used to calibrate the orientation of the lattice. The different colors were used to represent the positive and negative values of differential charge densities.

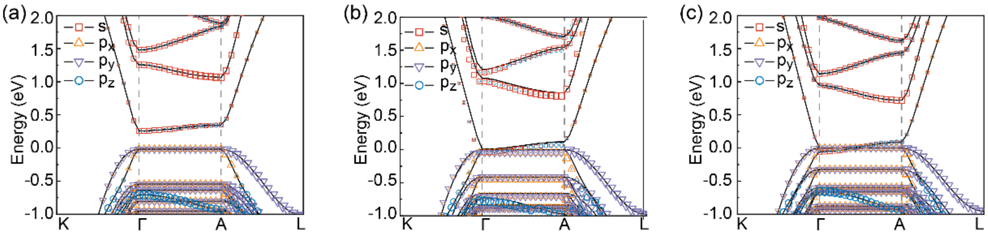

Figure S3 (**a**), (**b**) and (**c**) shown band structures between K and L points of InN_2/GaN_{10}, InN_3/GaN_9 and InN_4/GaN_8, respectively.

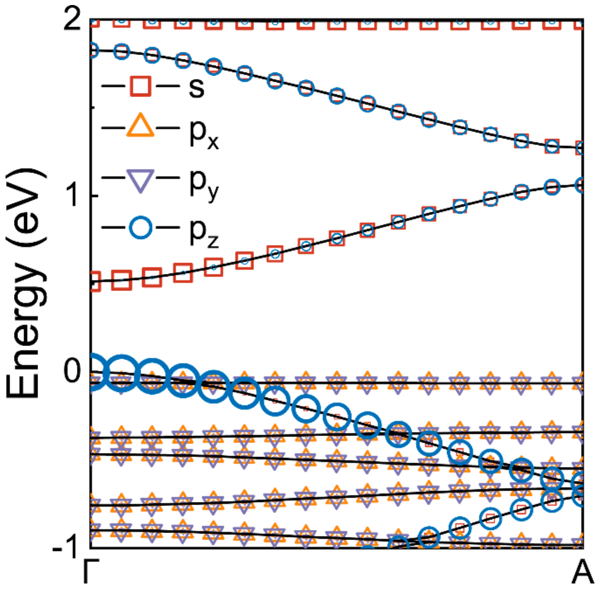

Figure S4 The electronic band structure of InN_1/GaN_7 under 8% tensile strain.

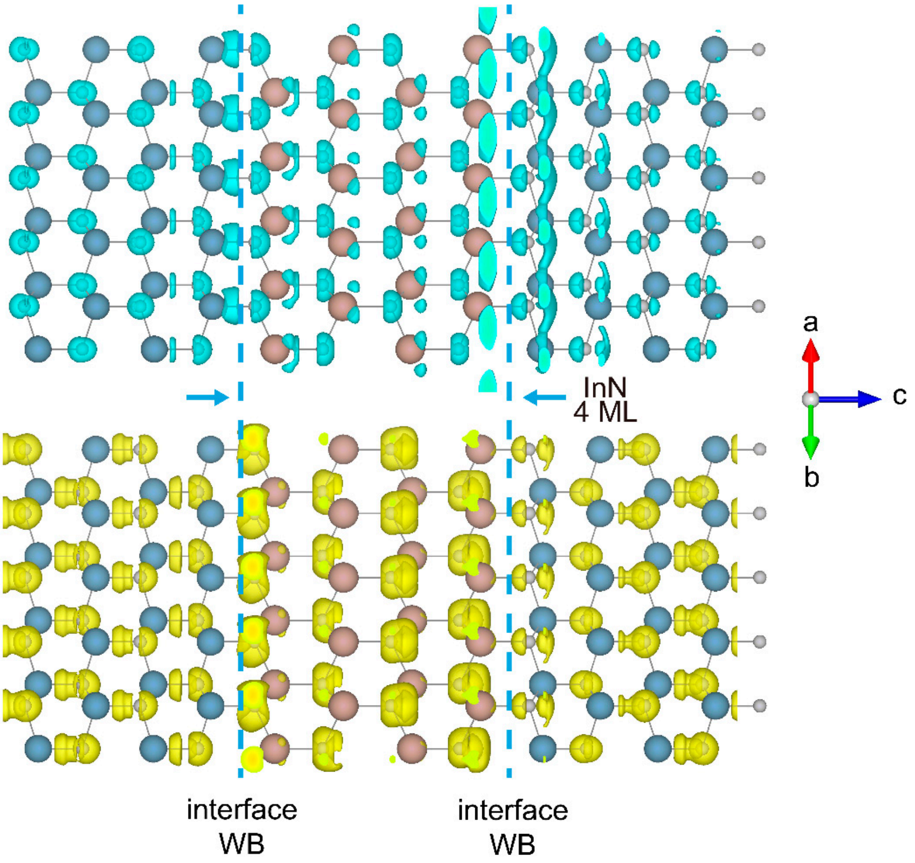

Figure S5 Distributions of injected electrons and holes. Blue and yellow surfaces represented contour planes of electrons and holes with charge density of 0.01 e/Å^3. The InN and GaN areas were divided by the blue dashed lines.
